# Caregiver Training in Mindfulness-Based Positive Behavior Supports (MBPBS): Effects on Caregivers and Adults with Intellectual and Developmental Disabilities

**DOI:** 10.3389/fpsyg.2016.00098

**Published:** 2016-02-09

**Authors:** Nirbhay N. Singh, Giulio E. Lancioni, Bryan T. Karazsia, Rachel E. Myers

**Affiliations:** ^1^Department of Psychiatry and Health Behavior, Medical College of Georgia, Augusta UniversityAugusta, GA, USA; ^2^Department of Neuroscience and Sense Organs, University of BariBari, Italy; ^3^Department of Psychology, The College of WoosterWooster, OH, USA; ^4^WellStar School of Nursing, Kennesaw State UniversityKennesaw, GA, USA

**Keywords:** Mindfulness-Based Positive Behavior Support, MBPBS program, staff training, physical restraints, staff stress, staff turnover, benefit-cost analysis

## Abstract

Caregivers often manage the aggressive behavior of individuals with intellectual and developmental disabilities that reside in community group homes. Sometimes this results in adverse outcomes for both the caregivers and the care recipients. We provided a 7-day intensive Mindfulness-Based Positive Behavior Support (MBPBS) training to caregivers from community group homes and assessed the outcomes in terms of caregiver variables, individuals’ behaviors, and an administrative outcome. When compared to pre-MBPBS training, the MBPBS training resulted in the caregivers using significantly less physical restraints, and staff stress and staff turnover were considerably reduced. The frequency of injury to caregivers and peers caused by the individuals was significantly reduced. A benefit-cost analysis showed substantial financial savings due to staff participation in the MBPBS program. This study provides further proof-of-concept for the effectiveness of MBPBS training for caregivers, and strengthens the call for training staff in mindfulness meditation.

## Introduction

Individuals with intellectual and developmental disabilities (IDD) commonly exhibit aggressive behaviors (Jahoda et al., 2013). The prevalence of aggression in this population varies widely, ranging from about 7 to 50%, depending on the population sampled, the sample size, level of functioning of the sample, the definition of aggression, institutional versus community sample, and the sampling method used (Emerson et al., 2001; Tenneij et al., 2009). Within this population, individuals with certain syndromes, such as Prader-Willi, Angelman, and Fragile X, appear to have a higher risk of engaging in aggression than others, such as those with Down and Williams syndromes (Powis and Oliver, 2014). In addition, autism spectrum disorders, communication deficits, impulsivity, and stereotypic behaviors appear to be associated with high levels of aggression. Furthermore, those individuals who engage in aggressive behaviors appear to have a higher risk for mental health problems than those who do not (Crocker et al., 2013). Thus, it is not surprising that much attention has been paid to developing effective interventions for aggression in this population (Didden et al., 2016).

Positive behavior support (PBS) appears to provide one of the best approaches to managing and controlling the aggressive behavior of individuals with IDD in the real world as opposed to in experimental studies (LaVigna and Willis, 2012). PBS is designed to decrease an individual’s problem behaviors by teaching new skills, modifying the environment where the problem behaviors occur, and enhancing quality of life. The core features of PBS include, “(1) a focus on valued outcomes as defined in collaboration with consumers; (2) the integration of behavioral, biomedical, and social science; (3) the selection and use of empirically validated procedures; and (4) the promotion of systems change as necessary to sustain positive outcomes” (Lucyshyn et al., 2015, p. 3). However, despite its proven effectiveness, PBS is not generally used in community settings across many countries, including the US and UK (Emerson et al., 2000). There has been much speculation in the research literature as to the reasons for this, including the finding that it is fairly labor-intensive and community staff are not adequately trained to implement behavioral interventions, including PBS programs (Allen et al., 2005). Thus, training in the basic principles and implementation of PBS has been seen as a priority if effective behavioral strategies are to be routinely used by staff in community settings for managing aggressive behavior (MacDonald, 2016). In the absence of such training, it is evident that staff often resort to the use of aversive or restrictive procedures (Deveau and McGill, 2014) and/or the use of psychotropic medication, despite its limited effectiveness in treating aggressive behavior (Tyrer et al., 2008; Singh et al., 2011a).

Regardless of the training provided to staff, they experience high levels of stress due to the challenging behaviors of individuals with IDD (Hastings, 2002; Hensel et al., 2012). This stress often leads to negative outcomes, including increased staff turnover (Hatton et al., 2001), decreased satisfaction with their jobs (Crawford et al., 2010), and lower quality of interactions with their care recipients (Schuengel et al., 2010). These findings have led to a number of research groups developing training programs focused on ameliorating psychological distress in support staff for individuals with IDD. For example, Noone and Hastings (2009, 2010) evaluated the effectiveness of Promotion of Acceptance in Carers and Teachers (PACT) in increasing resilience of support staff and reported a significant decrease in psychological distress even when there was a slight increase in occupational stress. PACT is based on Acceptance and Commitment Therapy (Hayes et al., 1999), and included acceptance (i.e., a willingness to experience the present moment without defense), mindfulness and values clarification components. In another study, Brooker et al. (2013) reported on the effectiveness of the Occupational Mindfulness (OM) program that was developed specifically to decrease stress and enhance psychological well-being as well as job satisfaction. The OM program includes core mindfulness practices (e.g., mindfulness of breathing, body scan meditation, and mindful stretching, sitting and walking), aspects of positive psychology, such as signature strengths (Seligman, 2002), and a number of cognitive therapy exercises.

Another approach has been to combine a staff occupational stress reduction program with training in techniques for managing the challenging behaviors of the care recipients. For example, Singh et al. (2009) added mindfulness-based training for support staff that had been provided behavioral training and assessed the effects of the combined approach on both staff and client behaviors. Results showed systematic decreases in aggressive incidents, as well as decreases in injuries to staff and peers. In addition, staff use of physical restraints and emergency medications for aggression and destructive behavior decreased dramatically. Singh et al. (2009) suggested that increasing the mindfulness of staff might have decreased their use of restrictive procedures with individuals with IDD. Indeed, Brooker et al.’s (2014) replication of these results, with a different set of mindfulness-based procedures, suggests that the mindfulness-based procedures may be the key components responsible for the behavior change in both the staff and the individuals with IDD.

In a study in which a mindfulness-based program was integrated with instruction in PBS, Singh et al. (2015) evaluated the effectiveness of Mindfulness-Based Positive Behavior Support (MBPBS) in staff use of restrictive procedures, as well as changes in staff and peer injuries. In addition, they evaluated the effectiveness of the MBPBS program in reducing staff stress and turnover, and investigated the benefit-cost of this program. The results showed that (a) staff stopped using physical restraints within a few weeks of MBPBS training, (b) staff and peer injuries were reduced to zero, and (c) there was a significant reduction in staff stress and zero staff turnover. In addition, the benefit-cost analysis showed substantial financial savings due to staff participation in the MBPBS training program. This study indicated that MBPBS might be an effective intervention for both staff stress and for managing the individuals’ aggressive behavior, as well as in providing financial savings to the service providers of individuals with IDD who engage in challenging behaviors.

The initial study by Singh et al. (2015) evaluated the effects of the MBPBS program with only nine staff and three individuals with intellectual disabilities who engaged in aggressive behaviors. Furthermore, because the study used a multiple-baseline design across group homes, the results while internally valid lacked external validity. Thus, the primary aim of the present proof-of-concept quasi-experimental study was to further evaluate the effectiveness of providing MBPBS training to support staff in reducing their use of restrictive procedures (e.g., physical restraint) and to assess resulting changes in staff and peer injuries. An additional aim was to provide confirmatory data on the effectiveness of MBPBS training in reducing staff stress and turnover, and to assess its cost effectiveness.

## Materials and Methods

### Ethics Statement

All training procedures in the study were in accord with the 1964 Helsinki declaration and its later amendments or comparable ethical standards. Informed consent was obtained from all individual participants included in the study.

### Participants

#### Staff

The participants were 33 direct care staff from five community group homes (i.e., six or seven staff per group home), with each home providing services to three or four adult individuals with developmental disabilities. They constituted the entire staff assigned to the morning and afternoon shifts at the five group homes. These staff were responsible for supervising the individuals in activities of daily living (ADL) including getting out of bed, toileting, bathing, dressing, grooming, eating, and getting ready for work or day programs, as needed. In addition, they assisted the individuals to do their own shopping, prepare their own meals, do their housekeeping and the laundry, manage their medications, make phone calls, travel on their own, and handle their finances. Each group home had two or three individuals who exhibited aggressive behaviors, although all residents were known to periodically engage in some form of verbal or physical aggression. All group home staff, including the 33 participating in the MBPBS training, had received new employee orientation training when they were initially employed in the community group homes and received a range of in-service training during their employment, including basic training in behavior management principles. In addition, because they were employed in group homes informally known as “behavior” homes, they received in-service training by behavior analysts whenever they were required to implement new PBS plans.

#### Individuals with IDD

Eighteen adults with IDD, 12 men and 6 women, lived in the five “behavior” group homes. Three group homes had four men each and two group homes had three women each. Their group home staff had been selected to receive the MBPBS training because 12 of the 18 individuals had behavior plans to manage their challenging behaviors, and the staff were experiencing some difficulties in maintaining and increasing the individuals’ socially acceptable behaviors. No individual was on one-to-one supervision by a support staff because of behavior problems, but all 18 individuals required support and assistance from staff for clinical and safety reasons.

Socio-demographic data on both the support staff and individuals with IDD are presented in **Table 1**. The service provider, group home staff, and institutional review committee approved the MBPBS training and data collection. The individuals with IDD did not receive any new, programmed behavioral interventions as a result of their group home staff being trained in the MBPBS program.

**Table 1:** Socio-demographic characteristics of the staff and individuals in the five group homes.

	Staff	Individuals with IDD
Number of participants	33	18
Mean age (years)	39	26
Age range (years)	19–49	18–37
Gender		
Male	17	12
Female	16	6
Average level of intellectual functioning	na	Mild
Number of individuals on psychotropic medications	na	12
Number of individuals with psychosis	na	12

*na, not applicable.*

### Procedure

#### Experimental Design

We used a quasi-experimental research design, generally known as one-group pretest–posttest design (Thyer, 2012). This quasi-experimental design provides a reasonable method for assessing the effects of MBPBS training on staff behavior and concomitant changes in the behavior of the clients in their care, as well as benefit-cost analyses with regards to training outcomes.

#### Pretraining

By policy and practice, the group home staff were required to document at point of contact whenever they used physical restraints for behavioral control with an individual in their care. In addition, they documented all instances of staff and peer injuries in the group home incident logs. For comparison purposes, retrospective data were obtained for 28 weeks and prospective pretraining data were monitored for 12 weeks (i.e., 40 weeks in total). No programmed intervention or training was instituted during pretraining.

#### Training

The training program was the same as reported by Singh et al. (2015). On the first day of Week 1, the staff were taught three basic meditations during a 1-day training. Training was provided in a group format with all 33 caregivers. They received the same instructions on the fundamentals of meditation posture for Samatha meditation as in Singh et al. (2015, p. 929): “... sit comfortably with a straight spine, without slouching or stretching the shoulders; head tilted slightly forward; eyes slightly open; tip of the tongue lightly touching the upper palate; right hand resting over the left hand on the lap, with thumbs just touching; and breathing evenly (Buksbazen, 2002). They were taught to focus on their breathing, without deliberately changing the length of each breath. They learned to count an inhalation and exhalation as one breath until they reached 10 breaths, before restarting the counting cycle. They were taught to simply observe their discursive thoughts and emotions, without interacting with them or trying to suppress them. That is, they were required to focus their awareness on whatever took place in their mind without judgment or engagement. Samatha meditation is the foundational meditation that provides the practitioner with the stability of mind on which to build all other meditation practices. In addition, they were taught Kinhin and Insight meditations (Buksbazen, 2002; McDonald, 2005). Kinhin is a walking meditation that enables a person to be in the present moment while walking slowly and mindfully. Vipassanā (Insight) meditation is used to gain insight into the true nature of reality through mindfulness of breathing, thoughts, feelings and actions (Shonin et al., 2015). Following the first day of training, all support staff were instructed to develop a personal meditation practice, beginning with a few minutes each day and incrementally increasing it until they reached between 20 and 30 min of daily practice. Finally, they were required to log their daily meditation practice.”

The staff practiced the three foundational meditations (i.e., Samatha, Kinhin, and Insight) daily for four weeks. During the 5th week, they received the 5-day intensive MBPBS training. As during the 1-day training, the 5-day training was provided in a group format. The training included instructions on and practice of the Four Immeasurables (lovingkindness, compassion, joy, and equanimity), the Three Poisons (attachment, anger, and ignorance), Shenpa and Compassionate Abiding, and Meditation on the Soles of the Feet (Kyabgon, 2004; Chödrön, 2007, 2010; Kongtrül, 2008; Singh et al., 2011b). In addition, staff were given explicit instructions on how to use PBS within the context of mindfulness practices. The PBS component of the instructions was informed by the existing literature on staff training in PBS (see MacDonald, 2016). That is, by the end of the 5-day training the staff were expected to be familiar with the following five components of standard PBS plans: setting event strategies, preventive strategies, teaching strategies, consequence strategies, and quality of life outcomes. In addition, they were given instructions on mindful observation of the individual’s behavior, mindful communication (with a focus on mindful prompting and feedback), mindful pause between requests and prompts, and use of reinforcement contingencies that focused on the rate, quality, magnitude, delay, and specificity of the reinforcement delivered by the support staff (Singh et al., 2016).

The staff translated their newly acquired MBPBS skills into daily practice from Week 6 for four weeks. They continued the meditation practices from the 1-day training, together with the MBPBS meditations and practices from the 5-day intensive training. On the first day of Week 10, the caregivers met with the trainer for the final 1-day training to review their meditation practices and use of MBPBS procedures, and to provide feedback on the training program. They were given no new instructions in MBPBS, but any questions they had about the mindfulness-based practices and the PBS techniques were fully answered by the trainer. **Table 2** presents the MBPBS program and a brief outline of each day’s training. Outcome data continued to be collected during the post-MBPBS phase, which lasted 40 weeks. That is, data were collected during the 40-week pre-MBPBS phase and the 40-week post-MBPBS phase that included the 10-week MBPBS training period and 30 weeks thereafter.

**Table 2:** Brief outline of the 7-Day MBPBS Program.

**Day 1** (First 1-day training)	Samatha meditation Kinhin meditation Insight meditation Five hindrances—sensory desire, ill will, sloth and torpor, restlessness and remorse, and doubt Daily logs and journaling

**Day 2** (First day of 5-day intensive training)	Review of meditation practice Introduction to the Four Immeasurables (*Brahmavihara*: *metta*—lovingkindness; *karuna*—compassion; *mudita*—empathetic joy; *upekkha*—equanimity) Equanimity meditation Beginner’s mind Applications to PBS practice

**Day 3**	Review of Day 2 instructions and practices Further instructions on the Four Immeasurables Equanimity meditation Lovingkindness meditation Being in the present moment Applications to PBS practice

**Day 4**	Review of Days 2 and 3 instructions and practices Further instructions on the Four Immeasurables Equanimity meditation Lovingkindness meditation Compassion meditation The three poisons—attachment, anger, and ignorance Applications to PBS practice

**Day 5**	Review of Days 2–4 instructions and practices Further instructions on the Four Immeasurables Equanimity meditation Lovingkindness meditation Compassion meditation Joy meditation Attachment and anger—shenpa and compassionate abiding meditations Applications to PBS practice

**Day 6**	Review of Days 2–5 instructions and practices Review and practice samatha, Kinhin, and Insight meditations Review of the Four Immeasurables Practice equanimity, lovingkindness, compassion and joy meditations Attachment and anger—meditation on the soles of the feet Review of applications to PBS practice Review of the MBPBS training program

**Day 7** (Second 1-day training)	Review of the meditation instructions and practices (daily logs) Review and practice samatha, Kinhin, and Insight meditations Review of the Four Immeasurables Practice equanimity, lovingkindness, compassion, and joy meditations Emotion regulation and anger—meditation on the soles of the feet Applications to PBS practice Review of the 7-day MBPBS training program

### Training Adherence

We recorded attendance of the 33 staff at the training sessions and each participant completed daily logs of meditation practices from pretraining to the end of the post-MBPBS training phase (i.e., 40 weeks). All 33 staff attended and fully participated in all seven training days. The daily logs showed that they initially engaged in meditation practice daily for a few minutes, first thing in the morning or last thing at night, during the pretraining phase until they were meditating between 15 and 20 min a day. They further increased their meditation sessions until they were sitting, using the Burmese posture (Buksbazen, 2002) or on a straight-back chair, for up to 30 min a day by the 20th week of post-MBPBS training. All support staff were able to maintain their daily meditations, on average, up to 30 min each day by the end of the post-MBPBS phase. On average, the staff meditated for 82% (range = 69–94%) of the days, for between 20 and 36 min, during the post-MBPBS training phase.

### Trainer

The trainer was an experienced behavior analyst at the BCBA-D level, with over 35 years of hands-on experience in developing and implementing behavior support plans. In addition, the trainer had a 40-year personal meditation practice and experience in the mindful delivery of services in behavioral health. Some training sessions were videotaped and five randomly selected segments of 15-min each from each day of the 7-day training (i.e., 35 training segments) were rated for fidelity of training by another qualified trainer. The fidelity of the MBPBS training was rated at 100%.

### Measures

#### Events

Staff documented the use of each instance of physical restraints, as required by policy in group homes. Physical restraints were defined as brief physical holds that staff administered when there was imminent danger of physical harm to an individual, peers or staff, and the behavior could not be controlled with verbal redirection. In addition, staff were required to document each instance of staff and peer injury caused by a resident in the five group homes. Staff injuries were defined as physical injuries to staff regardless of whether the staff was responding to the individual who was aggressive towards another individual or the individual was aggressive towards a staff member. Peer injuries were defined as physical injuries to peers by another individual during a physical altercation. Injuries were documented as physical injuries if they required first aid or further medical treatment. Data on events (use of physical restraints, staff injury, peer injury) were recorded immediately following each event at point of contact by the staff involved or staff who witnessed peer injury.

A trained independent observer was used to collect event data on the use of physical restraints, and staff and peer injuries, one day a week in each of five group homes, for 12 weeks during pre-MBPBS training and 40 weeks during post-MBPBS training for assessing the reliability of the staff recording the same events. On average, the independent observer collected data on the three events for 6 h, 1 day a week, at each group home for the duration of the study. No reliability data were available for the first 28 weeks of pretraining because the data were accessed retrospectively. An agreement was defined as the independent observer and the staff recording an instance of the use of physical restraint, staff injury, or peer injury as occurring at about the same time (i.e., within ± 1 min). Percent inter-rater agreement was calculated by dividing the total number of agreements by the total number of events recorded by the staff for each variable (i.e., physical restraint, staff injury, and peer injury) and multiplying by 100. The inter-rater agreement for all three events ranged from 97 to 100%, with a mean of approximately 99.5%. The few disagreements between the independent observer and the staff routinely recording the events were not in terms of whether an event occurred or not, but the exact time the event occurred.

#### Staff Stress

We used the Perceived Stress Scale-10 (PSS-10; Cohen et al., 1983) to measure staff’s perception of their stress at three time points: on the last day of the pre-MBPBS training; last day of MBPBS training (i.e., on the second 1-day training); and the last day of data collection (last day of post-MBPBS training in Week 40). The PSS-10 provides an index of the degree to which people perceive their lives as stressful and indicates how often they have found their lives to be unpredictable, uncontrollable, and overloaded in the last month. The staff responded to 10 questions on a 5-point scale, ranging from 0 (never) to 4 (very often). Responses were summed to create a psychological stress score, with higher scores indicating greater psychological stress. The PSS-10 has adequate psychometric characteristics (Cohen and Williamson, 1988).

#### Staff Turnover

We obtained staff turnover data from the Human Resources Department of the service provider. Staff turnover was defined as a staff member leaving the employment of the provider agency. We accessed staff turnover data only from the five group homes involved in the training and only for the first and second shift staff. Staff turnover data were obtained only for the 40 weeks of pre-MBPBS training and 40 weeks of post-MBPBS training. The reasons for leaving the employment of this service provider were also recorded.

#### Cost Effectiveness Data

We obtained cost data from the service provider’s Finance Department for (1) lost days of work due to staff injury, (2) days requiring temporary staff, (3) number of staff needing medical/physiotherapy care due to injury, (4) number of staff resigned due to staff injury and rehiring costs, (5) number requiring new employee training and training costs, (6) MBPBS training costs, and (7) cost of temporary staff required during MBPBS training. All costs were included regardless of whether the costs were incurred by the service provider or by the state Workers’ Compensation. We obtained cost data only for the 40 weeks of pre-MBPBS and 40 weeks of post-MBPBS training.

### Data Analyses

Multiple approaches to data analysis were adopted. First, changes in use of physical restraint, staff injuries, and peer injuries from the 40 weeks during pre-MBPBS training to the 40 weeks post MBPBS training were assessed with paired samples *t*-tests. Within each training period (pre and post), we then regressed physical restraint, staff injuries, and peer injuries on week of assessment to calculate the within-training period slope. This calculation enabled us to test the extent to which slopes within training periods differed from zero (e.g., were staff gradually using more, or less, physical restraint across the 40-week pre-MBPBS period?). In addition, repeated measures ANOVAs were used to analyze changes in perceived stress across the three timepoints of assessment (pre-MBPBS training, first post-MBPBS training, and second post-MBPBS training).

## Results

**FIGURE 1:**
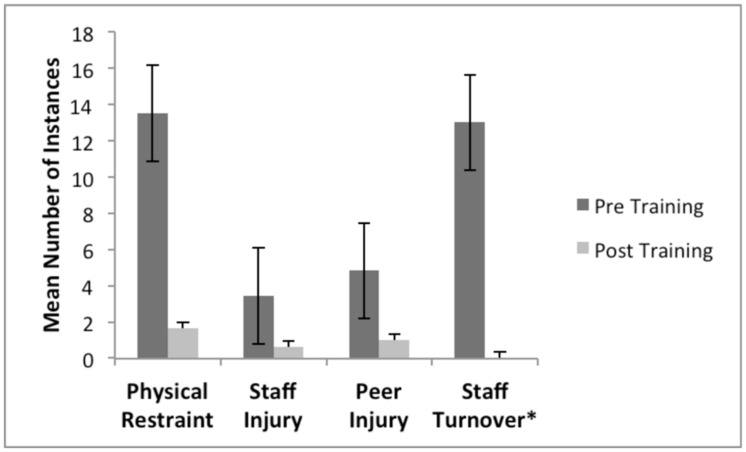
**Overall frequency of episodes of staff use of physical restraints, staff injury, peer injury, and staff turnover during Pre-MBPBS training and Post-MBPBS training phases.** ^∗^Staff turnover counts related to stress/injury and not all staff turnover.

The overall outcomes for staff use of physical restraints, staff injury, peer injury, and staff turnover are presented in **Figure 1**. Detailed analyses for each of these variables are presented below.

Weekly frequency of staff use of physical restraints decreased significantly from the 40-weeks during pre-MBPBS training (*M* = 14.00, *SD* = 3.31) to the 40-weeks during post-MBPBS training (*M* = 2.00, *SD* = 2.53), *t*(39) = –18.20, *p* < 0.001 (Cohen’s *d* = 5.83). Weekly injuries to staff decreased significantly between the pre-MBPBS training (*M* = 3.00, *SD* = 2.61) and post-MBPBS training (*M* = 1.00, *SD* = 1.06), *t*(39) = –7.44, *p* < 0.001 (Cohen’s *d* = 2.38). In addition, the change in weekly peer injuries from pre-MBPBS training (*M* = 5.00, *SD* = 3.14) to post-MBPBS training (*M* = 1.00, *SD* = 1.39) was statistically significant, *t*(39) = –7.67, *p* < 0.001 (Cohen’s *d* = 2.46).

The series of correlation analyses revealed that there were no significant correlations between week of assessment within phase (pre- or post-MBPBS Training) and use of restraint, staff injuries, or peer injuries (all 6 *p*-values > 0.05). This revealed that, within phase, there were no significant changes across time.

**FIGURE 2:**
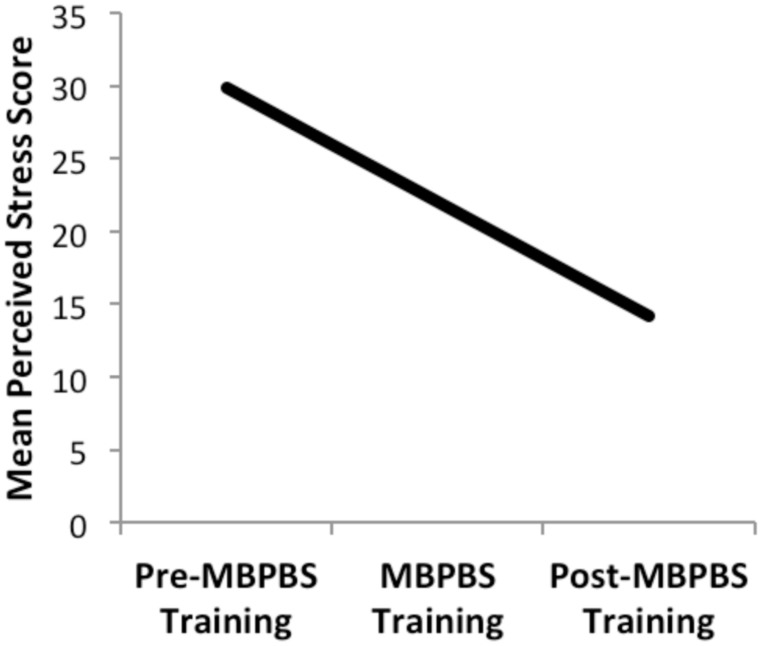
**Ratings of staff perceived stress on PSS-10 during the three phases of the study: Pre-MBPBS training, MBPBS training, and Post-MBPBS training.** Note that higher scores indicate greater psychological stress.

The support staffs’ overall perceived stress ratings are presented in **Figure 2**. Overall, there was 26.37% decrease in perceived stress from the last day of pretraining (Time 1) to the last day of the 7-day training (i.e., first post-training, Time 2), 35.67% decrease from the last day of the 7-day training (Time 2) to the last day of data collection in week 40 (i.e., second post-training, Time 3), and 52.64% decrease from the last day of pretraining (Time 1) to the last day of data collection in week 40 (Time 3). The change in perceived stress ratings across timepoints was statistically significant, *F*(2,64) = 417.56, *p* < 0.001; η^2^ = 0.93. *Post hoc* comparisons (Bonferroni) revealed that assessments at all three timepoints differed significantly from each other. That is, stress was highest at pretraining (*M* = 29.88, *SD* = 3.85). Reported stress by the end of the 7-day MBPBS training was significantly lower (*M* = 22.00, *SD* = 4.02), and reported stress at the end of the 40 weeks of post-MBPBS training was again significantly lower (*M* = 14.15, *SD* = 4.40).

In terms of staff turnover, 13 staff resigned during the 40 weeks prior to the beginning of the MBPBS training due to injury and stress incurred during this period. Another three staff resigned during the pre-MBPBS training period due to personal reasons (i.e., two changed jobs for higher pay; one retired). No staff member resigned during the post-MBPBS training period due to injury or stress. Two staff did resign during the post-MBPBS phase—one because of late stage pregnancy and the other due to his wife being promoted to an out-of-state job location. The difference between turnover due to injury and stress during the pre-MBPBS training versus post-MBPBS training phases was statistically significant, $\chi_{\left(1\right)}^{2}$ = 11.08, *p* < 0.001.

**Table 3** presents the cost effectiveness data. When compared to the 40 weeks of pre-MBPBS training, the number of lost days of work was reduced by 95% during the 40 weeks of the post-MBPBS training period for a savings of $52,752.00. This also resulted in an additional savings of $52,752.00 for the same period in terms of cost of hiring temporary staff for the lost days of work. The cost of medical and physical rehabilitation therapy services for the 16 injured staff during the 40 weeks of pre-MBPBS training was $384,000.00 compared to $0.00 during the post-MBPBS training period because no staff were injured. Additional costs were incurred during the pre-MBPBS training period with regard to rehiring ($2,600.00) and new employee training ($3,640.00). Furthermore, additional costs incurred during the post-MBPBS training included training costs ($22,500.00) and the cost of providing alternate staff for those who were in the 7-day training ($25,872.00). Overall, when compared to the pre-MBPBS training period, there was a savings of $447,372.00 for an equivalent period during post-MBPBS training; that is, a savings of 89.27%.

**Table 3:** Comparative costs for 40 weeks Pre-MBPBS training versus 40 weeks Post-MBPBS training for the seven human services variables.

	Cost variables		Cost	
	Pre-MBPBS	Post-MBPBS	Pre-MBPBS	Post-MBPBS
Lost days of work due to staff injury	495	24	$55,440.00	$2,688.00
Days requiring temporary staff	495	24	$55,440.00	$2,688.00
Number of staff needing medical and physical rehabilitation therapy	16	0	$384,000.00	$0.00
Number of staff resigned due to staff injury	13	0	$2,600.00	$0.00
Number of new staff NEO training	13	0	$3,640.00	$0.00
MBPBS training	–	–	$0.00	$22,500.00
Temporary staff during MBPBS training	–	–	$0.00	$25,872.00
Total Costs for the two time periods	–	–	$501,120.00	$53,748.00
**Total overall savings**	**$447,372.00**			

*NEO, new employee orientation.*

## Discussion

The results of the present study provide further evidence for the utility of the MBPBS program in helping staff better manage themselves and the individuals with IDD they provide services to. Our data showed a number of positive outcomes in terms of staff use of physical restraints for aggressive behavior, injuries to staff and peers, staff stress, staff turnover, and benefit-cost to the agency.

Following training in the MBPBS program, staff were able to reduce the use of physical restraints at a clinically and statistically significant level for controlling the aggressive behaviors of the individuals with IDD in their care. This finding provides further validation of the effectiveness of mindfulness-based training in helping staff to reduce the use of restrictive procedures. For example, Singh et al. (2009) showed that as training in mindfulness meditation progressed, the group home staff’s use of physical restraints and emergency medications decreased, with almost zero use of both interventions by the end of the study. These findings were essentially replicated by Brooker et al. (2014), who showed that staff trained in mindfulness meditations substantially reduced the use of emergency seclusions, *pro re nata* (prn) medications, and emergency chemical restraints for severe challenging behaviors of individuals with a disability. In the most recent study, Singh et al. (2015) reported the complete elimination of the use of physical restraints by group home staff following training in MBPBS.

When individuals with IDD are aggressive towards others, they injure staff and peers. Often, staff and peers need medical attention for their injuries that go beyond first aid. In the present study, there were statistically significant reductions in staff and peer injuries correlated with mindfulness-based training. Similar reductions in staff and peer injuries were reported by Singh et al. (2009, 2015). These are clinically significant findings because peer injuries often lead to increased behavioral altercations among the peers, produce fear in the victims of the aggression, and generally negatively impact the quality of life for both the aggressor and the victim. Furthermore, staff injuries often result in increased staff stress and staff turnover, which in turn negatively affects client services, and negatively impacts the health and wellness of the staff (Hastings, 2002). In the present study, there were statistically significant reductions in staff stress and staff turnover following staff training in the MBPBS program. There is ample research showing that mindfulness-based interventions substantially reduce stress in different populations, including staff and parents of individuals with IDD (Neece, 2014; Singh, 2014), and the current findings add to this evidence base.

Another fairly important finding in the present study was derived from the benefit-cost analyses. Costs were estimated in terms of the standard indicators used by service providers in the field of developmental disabilities—lost days of work, replacement staff, treatment related to work injuries, hiring and training of new staff, and training in the new technology. When comparable periods before and after MBPBS training were assessed in terms of benefit-cost to the service provider, there was a significant cost savings—just over 89%—due to the MBPBS training. This finding is in line with previous benefit-cost analyses of mindfulness-based procedures. For example, when adults with IDD were taught a mindfulness-based procedure to self-control their aggressive behavior, there was a 95.7% cost savings in terms of lost days of work and the cost of medical and rehabilitation treatment required by injured staff (Singh et al., 2008). In a subsequent study, when group home staff were trained on MBPBS, the additional costs directly related to the negative outcomes of managing the challenging behaviors of individuals with IDD was reduced by 87.75% (Singh et al., 2015). Given the fiscal savings, as well as health and wellness benefits to staff and clients, it appears that evidence from benefit-cost analyses provide a compelling argument for further evaluating the general benefits of training staff in MBPBS if they provide care for individuals with challenging behaviors.

There is a plethora of experimental research attesting to the effectiveness of behavioral interventions in the treatment of various challenging behaviors of individuals with IDD (see Singh, 2016). However, in the real world context of providing services to individuals with IDD, newly employed staff receive new employee training in behavioral methods for managing challenging behaviors, but the exigencies of providing care to multiple individuals under high demand situations often preclude the implementation of planned behavioral interventions with any degree of fidelity. Thus, providing staff with additional behavioral training during in-service courses does not reduce their stress and burnout because such training does not change how they deal with the requirements of the job itself. That is, it does not teach them to respond to the demands of their jobs in a different way. Perhaps what they need is training in how to change their *relationship* to the demands of the job rather than more knowledge about specific techniques for managing the challenging behaviors of the individuals with IDD in their care. Fortunately, one of the great strengths of a mindfulness-based approach is that it teaches staff how to respond differently to a demanding situation without trying to directly change it.

The results of the present study provide further support for this notion because the meditations included in the MBPBS program assist the staff to engage in personal transformation in terms of their relationship with life itself—how they respond to and accept life’s daily hassles, large and small. Tibetan Buddhist teachings emphasize that if there is any merit to one’s meditation efforts, the merits are shared with all sentient beings (Mingyur Rinpoche, 2014). Thus, when staff learn to meditate on a regular basis, the effects of their meditation have proximal and distal effects, not only on their own internal and external environments, but also on all sentient beings they come into contact with. In the context of this study, this would include the individuals with IDD and their peers, other staff and supervisors, and the service provider.

When staff meditate on a regular basis, their relationship to the individuals in their care changes from one of control to support. For example, the samatha (calm abiding or tranquility) meditation teaches the staff to rest their awareness on a moment-by-moment basis on the individual they are caring for, and then use mindfulness in a regulatory capacity to keep their attention on the individual, as well as to remember what they need to do to support the individual in his or her activities. The effects of the Four Immeasurables are that they have more equanimity as they go through their demanding work, they are more accepting of the individuals as they are—even of their challenging behaviors, and they share more lovingkindness, compassion and empathetic joy with the individuals in their care, as well as with their fellow staff and supervisors (Kyabgon, 2004).

The personal transformation in the staff that results from the meditation practice indirectly affects the individuals in their care, as evidenced in their behavioral interactions that gradually become more positive and less effortful (Bluth and Wahler, 2011). We suspect that what essentially happens is that the staff-client transactional pathway becomes increasingly positive, as both the staff and the individuals with IDD detect the subtle changes that occur in each other’s behavior and match the valence of their responses (Sameroff, 1995). In addition, some of the changes in staff behavior can be attributed to their enhanced awareness, derived from the mindfulness training, so that they can disengage themselves from their premature cognitive commitment to control the aggressive behavior of the individuals with physical restraints. The staff were taught to observe the individuals’ challenging behaviors with a beginner’s mind (Suzuki, 1970) that is empty of preconceived notions about its management. With a beginner’s mind, their meditation practice enabled them to wait, without judgment, until the appropriate response arose of its own accord (Kyabgon, 2004). While these are likely mechanisms of the observed changes in the behavior of the staff and of the individuals in their care, we do not want to overly speculate ahead of the data because this study was not designed to tease out the mechanisms involved.

There are obvious limitations of this study, with the primary one being the use of a quasi-experimental research design. While the design enables us to demonstrate change in staff and client behaviors, it does not allow us to make legitimate causal inferences because of the lack of a control group. Furthermore, the lack of a control training condition means that the possibility of a placebo effect cannot be ruled out. The data derived from the use of quasi-experimental research provide an indication of the effects that must eventually be tested in a randomized controlled trial. However, the replication of the effects across proof-of-concept studies strengthens the conclusions drawn in this study. Indeed, it is questionable whether a randomized controlled trial should be undertaken without data from several proof-of-concept studies to indicate that the new intervention is indeed likely to be effective. Another limitation is the non-random selection of the staff and the clients they served because this study focused on only those staff who worked in high demand situations. Whether similar results would be obtained with any group home staff, regardless of whether their clients had challenging behaviors, remains to be investigated. Another limitation is that the staff had varying levels of training in implementing traditional behavior plans. This variable could not be controlled, as behavioral training is mandatory for such staff.

There is the additional issue of how effectively staff can be trained to deliver the MBPBS training. Like other mindfulness-based programs, such as the Mindfulness-Based Stress Reduction (MBSR) program (Kabat-Zinn, 1990), MBPBS requires the trainer to have a long-standing personal meditation practice as well as being well-versed in applied behavior analysis and the principles and practice of PBS. Furthermore, many of the meditations used in MBPBS are derived from the Buddha’s teachings, although they can be taught in a secular format. However, there has been some disquiet about stripping the teachings from their Buddhist foundations (e.g., Amaro, 2015; Grossman, 2015; Monteiro et al., 2015; Purser, 2015; Shonin et al., 2015). Obviously, staff and clients do not have to believe in or follow Buddhism, but the person who wants to teach MBPBS must have a certain familiarity with these critical issues. The MBPBS training is fairly intensive, requiring 56 h of training followed up by personal practice. While this appears to be a demanding time commitment, it may not be any more than the cumulative amount of time staff currently spend in behavioral training alone.

Future studies should not only use more robust experimental designs, but also evaluate the efficacy and effectiveness of MBPBS against currently accepted practices (e.g., PBS alone, traditional behavior modification approaches, cognitive behavioral therapy), as well as the newer mindfulness-based and ACT procedures (e.g., OM, PACT). In addition, it would be important to establish the feasibility and acceptability of MBPBS practices in developmental centers and community-based settings. Furthermore, the breadth of outcome measures could be expanded to include other key indicators of service provision, such as special staffing requirements for aggressive individuals with IDD (e.g., 1-to-1 or 2-to-1 staffing) and effects on staff (e.g., compassion fatigue).

In sum, this study adds to the small but growing research that indicates mindfulness-based procedures can be effective in training staff to deliver services to individuals with IDD that does not require the use of restrictive procedures. Furthermore, these studies suggest that a disciplined practice of meditation enables personal transformation that results in much reduced stress in daily life. Finally, with specific reference to MBPBS, the accumulating proof-of-concept studies strongly indicate that the program may be effective in enhancing the overall quality of life of the staff as well as their clients, and result in financial benefits to the provider service agency.

## Author Contributions

NS: designed and executed the study; assisted with the data analyses; and wrote the first draft. GL: collaborated with the design and writing of the study. BK: analyzed the data and wrote the results. RM: collaborated on the data analyses, write up, and final editing of the manuscript.

## Conflict of Interest Statement

NNS is the developer of the MBPBS program. The authors declare that the research was conducted in the absence of any commercial or financial relationships that could be construed as a potential conflict of interest.
